# Interethnic diversity of *NAT2 *polymorphisms in Brazilian admixed populations

**DOI:** 10.1186/1471-2156-11-87

**Published:** 2010-10-05

**Authors:** Jhimmy Talbot, Luiz Alexandre V Magno, Cinthia VN Santana, Sandra MB Sousa, Paulo RS Melo, Ronan X Correa, Giuliano Di Pietro, Fabrício Rios-Santos

**Affiliations:** 1 Universidade Estadual de Santa Cruz, Laboratório de Farmacogenômica e Epidemiologia Molecular (LAFEM), Ilhéus, Bahia, Brazil; 2 INCT de Medicina Molecular, Faculdade de Medicina, Universidade Federal de Minas Gerais, Brazil

## Abstract

**Background:**

N-acetyltransferase type 2 (Nat2) is a phase II drug- metabolizing enzyme that plays a key role in the bioactivation of aromatic and heterocyclic amines. Its relevance in drug metabolism and disease susceptibility remains a central theme for pharmacogenetic research, mainly because of its genetic variability among human populations. In fact, the evolutionary and ethnic-specific SNPs on the *NAT2 *gene remain a focus for the potential discoveries in personalized drug therapy and genetic markers of diseases. Despite the wide characterization of *NAT2 *SNPs frequency in established ethnic groups, little data are available for highly admixed populations. In this context, five common *NAT2 *SNPs (*G191A*, *C481T*, *G590A*, *A803G *and G*857A*) were investigated in a highly admixed population comprised of Afro-Brazilians, Whites, and Amerindians in northeastern Brazil. Thus, we sought to determine whether the distribution of *NAT2 *polymorphism is different among these three ethnic groups.

**Results:**

Overall, there were no statistically significant differences in the distribution of *NAT2 *polymorphism when Afro-Brazilian and White groups were compared. Even the allele frequency of *191A*, relatively common in African descendents, was not different between the Afro-Brazilian and White groups. However, allele and genotype frequencies of *G590A *were significantly higher in the Amerindian group than either in the Afro-Brazilian or White groups. Interestingly, a haplotype block between *G590A *and *A803G *was verified exclusively among Amerindians.

**Conclusions:**

Our results indicate that ethnic admixture might contribute to a particular pattern of genetic diversity in the *NAT2 *gene and also offer new insights for the investigation of possible new *NAT2 *gene-environment effects in admixed populations.

## Background

Genetic functional polymorphisms of xenobiotic/drug metabolizing enzymes have been associated with pharmacotherapy response differences and disease risk susceptibility [1,2]. Special emphasis has been placed on a phase II metabolizing enzyme, N-acetyltransferase type 2 (Nat2, EC 2.3.1.5), a milestone in the pharmacogenetics field as one of the first enzymes to be associated as a cause of interindividual variation in drug metabolism [3]. Nat2 catalyzes a transfer of an acetyl group from the cofactor acetyl-coenzyme A (acetyl-CoA) to the amine nitrogen atom of aromatic amines and hydrazines [4]. This enzyme is important in the aromatic and heterocyclic amine conjugating reaction, preventing their metabolic activation into electrophilic intermediates that could initiate DNA damage and potentially induce carcinogenic mutations [5]. Moreover, Nat2 plays a role in the metabolism of different hydrazine and arylamine drugs, such as isoniazid and dapsone, both used in the treatment of *Mycobacterium *spp. infections [6,7].

The human *NAT2 *gene has an intronless open reading frame of 870 base pairs and is primarily expressed in the liver and intestines [8-10]. It has long been recognized that some single nucleotide polymorphisms (SNPs) in the *NAT2 *gene may change protein structure and/or stability and segregate in humans into "rapid", "intermediate" and "slow" acetylation phenotypes [11,12]. The effects of genetic polymorphism in the *NAT2 *gene on N-acetylation activity led to investigations of *NAT2 *SNPs as a promising genetic marker for disease risk, drug response therapy and/or adverse reactions to drugs [13]. For example, slow acetylators are at increased risk of peripheral neuropathies and systemic lupus erythematosus due to hepatotoxicity to isoniazid treatment, hypersensibility reactions to sulphonamides and poor tolerance to sulfasalazine and dapsone [14]. Conversely, some authors demonstrated increased risk of myelotoxicity induced by amonafide in rapid acetylators, probably due to the production of higher levels of toxic metabolites from drugs [15].

If genetic factors underlie disease risk, the distribution of susceptibility alleles may be influenced by ethnic diversity [16]. Consistent with this question, several studies have shown that frequencies of *NAT2 *SNPs differ among established ethnic groups [17]. Although the characterization of the frequency of *NAT2 *SNPs has been established in some ethnic groups, it is still necessary to investigate the distribution *of NAT2 *SNPs in populations characterized by a high degree of admixture. Brazilian populations are of particular interest since it was historically originated by Caucasian settlers, descendents of African slaves, and by Amerindians [18-20]. In this context, we investigated the frequency of five common *NAT2 *SNPs (*G191A*, *C481T*, *G590A*, *A803G *and G*857A*; *G191A*) and haplotype structure in a highly admixed population in Northeastern Brazil.

## Methods

### Subjects

All 183 individuals included in the current study were residents in the Ilhéus area, healthy blood donors at São José Hospital (Ilhéus, Bahia, Brazil) and reported at least 3 familial generations resident in Northeastern Brazil. Volunteers were randomly selected during a 6-month period and classified by self-reported ancestry into Afro-Brazilian, Amerindians (native Brazilian descendents of the Tupinamba tribe) or White. The Human Ethical Committee of Universidade Estadual de Santa Cruz approved the study and all volunteers gave their informed consent.

### Sample collection and genotyping

Peripheral blood was collected and genomic DNA isolation was performed from white blood cells using the FlexiGene DNA Kit (Qiagen, Boston, USA). *NAT2 *genotypes were determined using a modification of a polymerase chain reaction (PCR)-restriction fragment length polymorphisms (RFLP) assay [21]. Genomic DNA (100 ng) amplification was carried out in a 25 μL-reaction volume containing 10 mmolL^-1 ^Tris-HCl (pH 8.3), 50 mmolL^-1 ^KCl, 1.5 mmolL^-1 ^MgCl_2_, 0.2 mmolL^-1 ^of each deoxynucleotide triphosphate (dNTP), 0.2 μmolL^-1 ^of each oligonucleotide primer (Forward, 5'-GGCTATAAGAACTCTAGGAAC-3' and Reverse, 5'-AAGGGTTTATTTTGTTCCTTATTCTAAAT-3'), and 1.25U of Platinum Taq DNA polymerase (Invitrogen, California, USA). Thermal cycling conditions for the PCR were as follows: 5 min at 94°C, followed by 35 cycles of 94°C for 1 min, 55°C for 1 min, and 72°C for 1 min, with a final extension at 72°C for 5 min.

Following amplification, genotyping was performed using a RFLP assay to detect five different *NAT2 *SNPs: *G191A *(*rs1801279*), *C481T *(*rs1799929*), *G590A *(*rs1799930*), *A803G *(*rs1208*) and *G857A *(*rs1799931*). In this assay, PCR products (895 bp) were digested separately with *Msp*I, *Kpn*I, *Taq*I, *Bam*HI (Fermentas, Canada) and *Dde*I (New England Biolabs, USA) to detect a specific SNP (Table 1). All digestions were performed according to the manufacturer's recommendations. Digested PCR products were separated by electrophoresis on 2% agarose gels (Pronadisa, Madrid, Spain) for *Msp*I, *Kpn*I and *Bam*HI or 10% polyacrylamide gels for *Taq*I and *Dde*I (90-100 V for 120 min or 6 hours, respectively) with DNA molecular size markers (Invitrogen, California, USA). The amplified products were visualized with ethidium bromide staining under UV light.

**Table 1:** *NAT2 *SNPs used in this study.

SNPs	RFLP Enzyme	Aminoacid Substitution	Enzymatic Activity	Reference
***G191A*** (*rs1801279*)	*Msp*I	Arg64Gln	Reduced	[28,29]
***C481T*** (*rs1799929*)	*Kpn*I	-	Non-Altered	[28]
***G590A*** (*rs1799930*)	*Taq*I	Arg197Gln	Reduced	[28]
***A803G*** (*rs1208*)	*Dde*I	Lys268Arg	Non-Altered	[26]
G857A* (*rs1799931*)	*Bam*HI	Gly286Gln	Reduced	[28,30,31]

These levels are referents to the presence of a single SNP and If there is not anyone SNP in the 2 gene copies. * Substrate dependent

### Statistical analysis

Individual marker analysis comparing genetic and allelic frequencies among ethnic groups was performed using χ^2 ^tests. Multiple logistic regression analysis to evaluate ethnic influences on the polymorphism frequency and genetic associations were carried out using the Statistical Package for the Social Sciences v.15.0 (Chicago, IL, USA) and UNPHASED v.3.0.13 [22], respectively. We used default settings of HAPLOVIEW v.4.1 software [23] to evaluate pairwise linkage disequilibrium (LD) between the five SNPs, genotype deviation from Hardy-Weinberg equilibrium (HWE) and for association between haplotypes defined by block in comparison groups [23]. For an accurate type I error, we performed 1,000 permutations in each procedure test to estimate the global significance of the observed differences. The test computes the significance by counting the number of ways the data can be permuted to determine how unusual an observed outcome is. All tests were two-tailed and the *p *level of significance retained was 0.05.

## Results

### Allelic and Genotypic Associations

The sample was composed predominantly of Afro-Brazilians (68.31%), Whites (15.3%) and Amerindians (16.39%) (average age 34.3 ± 10.2 yrs, 71% males).

The allele and genotype frequencies of the *NAT2 *SNPs obtained from all individuals and in separate ethnic groups are summarized in Table 2. *481T *was the most frequent allele with 38.79% in the general population (33.9 - 43.3%) whereas the *191A *allele was less frequent in the three ethnic groups ranging from 5.0 and 10.7% (8.8% in total). No statistically significant differences were observed in the distribution of *NAT2 *polymorphisms when comparing Afro-Brazilian and White groups (Table 2). However, allelic and genotypic frequencies of *G590A *polymorphism were significantly increased in Amerindians when compared with other ethnic groups and remained statistically different after multiple testing corrections (Table 2), and multiple logistic regression analysis (OR = 3.714; 95%CI = 0.28-8.54; *p *= 0.040) (Table 3). Although homozygous carriers of the *A803G *mutant allele (*GG*) were not detected in Afro-Brazilian individuals in this study, this genotype was found in the White and Amerindian descendents with a frequency of 6.1 and 3.4%, respectively. Similarly, homozygous carriers of G857A wild allele (AA) were not found in the Amerindian group.

**Table 2:** Genotypic and allelic frequency of *NAT2 *gene in different ethnic groups.

*NAT2* SNP	Total	Afro-Brazilians	*χ*^2^	*p*^*1*^	Whites	*χ*^2^	*p*^*2*^	Amerindians	*χ*^2^	*p*^*3*^
***G191A***										
GG	158	104			23			27		
GA	22	20	1.075	*0.5842*	4	1.781	*0.4104*	3	1.207	*0.547*
AA	3	1			1			0		
G	28	228	0.202	*0.6593*	50	1.322	*0.2471*	57	0.942	*0.3061*
A	338	22			6			3		
***C481T***										
CC	72	41			11			12		
CT	79	60	2.565	*0.2774*	15	4.836	*0.089*	10	2.36	*0.3073*
TT	31	23			2			8		
C	223	142	1.466	*0.2221*	37	1.079	*0.2982*	34	0.007	*0.9338*
T	141	106			19			26		
***G590A***										
GG	92	77			19			10		
GA	67	46	1.298	*0.5224*	8	4.312	*0.1158*	14	9.943	***0.0069^b^***
AA	24	2			1			6		
G	251	200	0.005	*0.9405*	46	4.661	***0.0288^a^***	34	8.4	***0.0053^c^***
A	115	50			10			26		
***A803G***										
AA	107	64			15			17		
AG	73	61	3.662	*0.1603*	11	1.333	*0.5135*	12	0.954	*0.6207*
GG	3	0			2			1		
A	287	189	1.686	*0.1836*	41	0.117	*0.7316*	46	0.834	*0.3538*
G	79	61			15			14		
***G857A***										
GG	129	86			17			24		
GA	51	33	0.986	*0.6108*	10	3.391	*0.1835*	6	3.244	*0.1975*
AA	3	6			1			0		
G	309	205	0.419	*0.524*	44	2.750	*0.0964*	54	2.013	*0.1373*
A	57	45			12			6		

***p***: Genotype and allelic p-value for each marker, respectively. ***p***^***1***^, ***p***^***2 ***^and ***p***^***3***^: Association between Afro-Brazilians × Whites; Whites × Amerindians and Amerindians × Afro-Brazilians, respectively. ***a***, ***b ***and ***c***: *p*-value adjusted (10^3 ^permutations) = 0.06494, 0.006993 and 0.004995, respectively. ***χ***^***2***^: Chi-square tests. Significant association is indicated in bold.

**Table 3:** Logistic regression analysis for risk of *NAT2 *mutant allele carrier among ethnic groups

	Afro-Brazilians OR (CI 95%)	*p^1^*	Whites OR (CI 95%)	*p^2^*	Amerindians OR (CI 95%)	*p^3^*
***191A***	1.81 (0.50-6.54)	0.392	1.95 (0.42-9.08)	0.361	0.22 (0.28-1.76)	**0.0003**
***481T***	1.35 (0.59-3.06)	0.474	0.97 (0.33-2.78)	0.651	1.50 (0.25-8.71)	0.277
***590A***	0.33 (0.12-0.95)	0.317	0.27 (0.07-1.03)	0.056	3.71 (0.28-8.54)	**0.040**
***803G***	1.52 (0.67-3.47)	0.320	0.97 (0.33-2.82)	0.961	1.02 (0.35-2.98)	0.356
***857A***	1.73 (0.65-4.60)	0.273	2.56 (0.77-8.51)	0.126	0.39 (0.05-2.89)	**0.003**

Reference group is *G191*, *C481*, *G590*, *A803 *and *G857*, respectively. Odds Ratio (OR) and 95% Confidence Interval (CI) for each reference allele compared are shown. ***p**: p*-value. ***p***^***1***^, ***p***^***2 ***^and ***p***^***3***^. Association between Afro-Brazilians × Whites; Whites × Amerindians and Amerindians × Afro-Brazilians, respectively. Significant association is indicated in bold.

### Haplotypic Associations

**Figure 1:**
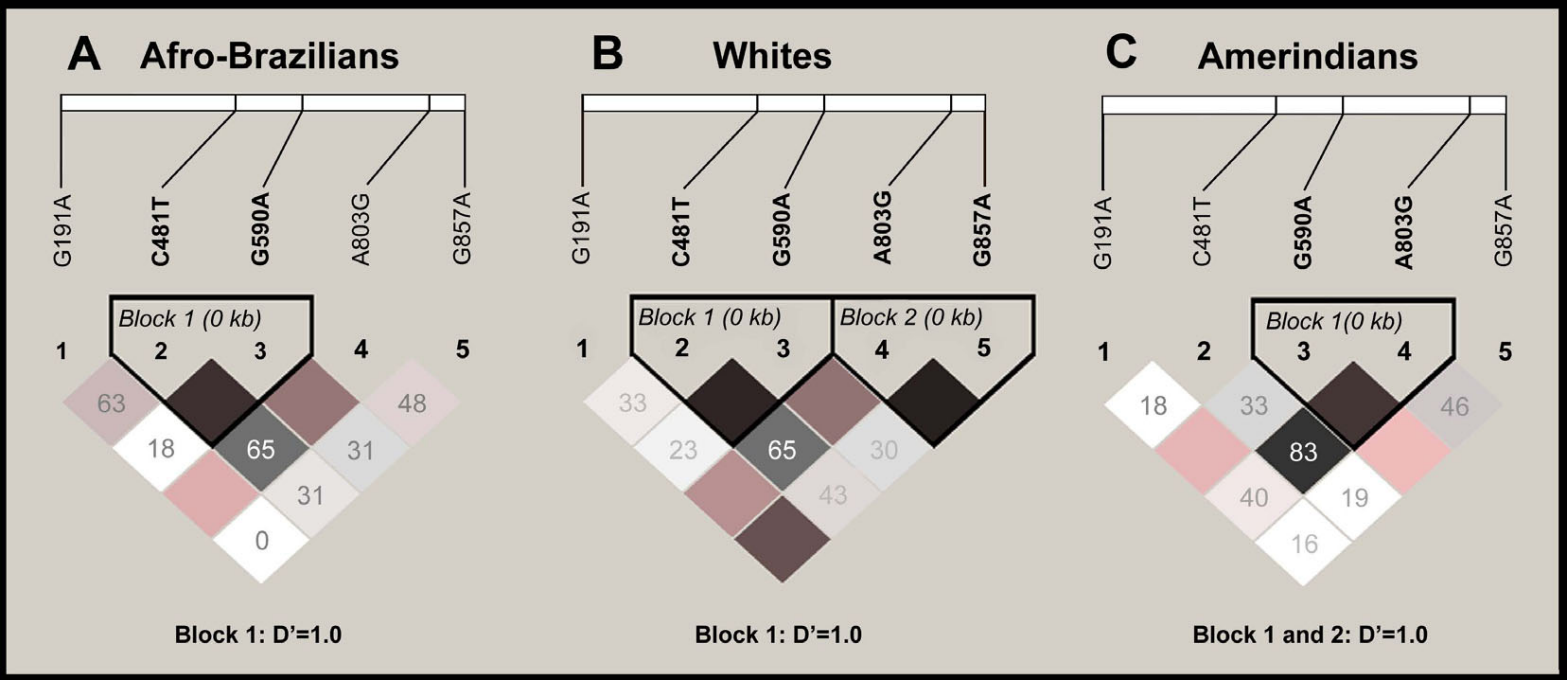
**LD block structure across *NAT2 *gene**. The upper panel shows the location of 5 polymorphisms in *NAT2 *gene and lower panel shows the output of Haploview. LD plot where in each square (with D' values written within the box) represent a pairwise linkage disequilibrium relationship between the two SNPs. Squares indicate statistically significant LD between the pair of SNPs as measured by the D' statistic. Darker colours of grey indicate higher values of D', up to a maximum of 1 and white squares indicate pairwise D' values of < 1 with no statistically significant evidence of LD. The blocks generated under confidence interval algorithm of Haploview are marked. A: Afro-Brazilians, B: Whites and C: Amerindians.

The sample was in Hardy-Weinberg equilibrium (p > 0.05) for the five SNPs (Table 4), as well as separately by ethnicity (data not shown). *NAT2 *SNP combinations were inferred from haplotype data. Linkage disequilibrium (LD) analysis in the general population revealed that the five common *NAT2 *SNPs had the weak D' as well as some low r^2 ^values. Haplotype blocks were constructed if D' between SNPs was 1.0. Using this criterion, significant differences in haplotype structure among the ethnic groups were observed (Figure 1).

**Table 4:** *NAT2 *haplotypes frequency in Brazilian ethnic groups.

Haplotype		SNPs (HWpval)					Haplotypes (n)				
	C.N.	*G191A* (*0.348*)	*C481T* (*0.613*)	*G590A* (*1.0*)	*A803G* (*0.303*)	*G857A * (*0.524*)	Total	Afro-Brazilians	Whites	Amerindians	
A	(NAT2*4)	G	C	G	A	G	45	33	9	6
B	(NAT2*C)	G	T	G	G	G	35	6	4	3
C*	(NAT2*6A/*6B)	**G**	**C**	**A**	**A**	**G**	**23**	**3**	**1**	**7**
D	(NAT2*11A/*11B)	G	T	G	A	G	23	5	2	2
**E**	(NAT2*6I/6J)	**G**	**C**	**A**	**A**	**A**	**11**	**2**	**1**	**-**
F	(NAT2*7A/*7B)	G	C	G	A	A	9	1	3	-
**G**	(NAT2*14A/*14B/*14H)	**A**	**C**	**G**	**A**	**G**	**8**	1	1	-
H	(NAT2*12)	G	C	G	G	G	6	1	1	-
I**	(NAT2*6E)	**G**	**T**	**A**	**A**	**G**	**5**	**-**	**-**	**3**
J	*(n.ds.)*	G	T	G	A	A	4	1	-	0
K	(NAT2*12C+857A)	G	T	G	G	A	4	1	-	1
**L**	(NAT2*14C/*14I)	**A**	**T**	**G**	**A**	**G**	**3**	**-**	**1**	**-**
**Slow Acetylator Haplotype**		**A**	**N**	**A**	**N**	**N**	**50**	29^+^	6^+^	11^+^

**HWpval**: Hardy-Weinberg equilibrium *p*-value for the whole sample. * and ** significant difference in frequency between Amerindians and Afro-Brazilians (***χ*^2^**: 6.517; *p *= 0.0107 and ***χ*^2^**: 5.539; *p *= 0.0186, respectively). C, E, G, I and L are slow acetylator haplotypes and are indicated in bold. '**-**': Absent or with frequency < 1%. '**N**': any nucleotide. '**+**': haplotypes with frequencies < 1% are included. **C.N**.: *Consensus Human Arylamine N-Acetyltransferase Gene Nomenclature *-http://louisville.edu/medschool/pharmacology/consensus-human-arylamine-n-acetyltransferase-gene-nomenclature; **(n.d.s)**: SNP combination not described

Additionally to the haplotype analysis according to LD pattern, another haplotype construction based on acetylator phenotype was performed using the most common *NAT2 *slow SNPs. Thus, this approach estimates a minimum percentage of slow acetylators and avoids misclassification of *NAT2 *haplotypes in accordance with official nomenclature. The *G191A *SNP, which leads to an amino acid change in position 64 of the Nat2 protein, produces an enzyme with reduced acetylation capacity [24,25]. The same functional phenomenon occurs with the *G590A *allele. In this context, we estimated that any haplotype comprising at least one of these alleles, *191A *and *590A*, should be theoretically treated as a slow acetylator. It is worthwhile to observe that the slow acetylator haplotypes are underestimated, since the *857A *allele may also generate the slow acetylator phenotype (NAT2*7A and NAT2*7B) [26].

Using this criterion, the haplotype distribution is demonstrated in Table 4. For practical purposes, the haplotype distribution was labelled from A to L in a sliding scale, correspondent to and in accordance with the human *NAT2 *nomenclature. Five slow acetylator haplotypes were found (C, E, I, J, and L), which were higher in Amerindians (44.9%) than in Afro-Brazilians and Whites (22.9% and 25.8%, respectively) (Table 4). Interestingly, we found some haplotypes in Amerindians that were not found in the other groups (data not shown). The most frequent haplotype among Afro-Brazilians and Whites was "A" (26.1 and 35.3%, respectively), whereas C was the most frequent haplotype among Amerindians (28.6%). Among all haplotypes, only the distribution of "C" and "I" was statistically different between Amerindians and Afro-Brazilians (p = 0.0107 and p = 0.0186, respectively) (Table 4). These two haplotypes are correspondent to NAT2*6 haplotype subgroups, ("C" = NAT2*6B; "I" = NAT2 *6E).

## Discussion

Ethnicity is an important variable that influences an individual's health in several ways, in particular increasing risks for the development of chronic diseases and unresponsiveness or adverse reactions to drug treatment [27]. The influence of the ethnic component in the distribution of *NAT2 *genetic polymorphism is well established. An example is the ethnic-specific *191A *allele, mainly identified in Africans (7-20%) and with lower frequencies in Euro-Caucasian groups (less than 2%) [28-31]. Another example is the *857A *allele, mainly identified in eastern Asians [32].

Following our initial purpose of investigating the frequency of *NAT2 *SNPs in a Brazilian admixed population, the five most common *NAT2 *SNPs known from published research were selected. Except for *C481T *SNP, which does not alter the Nat2 enzymatic function, the other four SNPs are associated with slow acetylator status and had high frequencies in our whole sample as well as among ethnic groups (Table 1 and 2). Although the *191A *allele has been described as relatively common in African populations but not in Caucasians, no significant difference was observed between both groups in this study (Table 2). Similar results were obtained for *G590A*. The ethnic similarity in the distribution of *NAT2 *SNPs observed in this study could be due to the high degree of admixture between Afro-descendents and Euro-Caucasian groups (mainly Portuguese settlers) that has occurred in Brazil over the centuries since colonization [33]. However, the bias from the self-reported ancestry classification method can not be totally excluded.

Interestingly, meaningful results from the distribution of *NAT2 *alleles in Amerindian descendents were observed. The *590A *allele was significantly more frequent in Amerindians (42.5%) than in White or Afro-Brazilian descendents (19.4% and 20.0%, respectively), even after permutation tests to decrease the risk of a type I error (Table 2). Multiple logistic regression analysis confirmed that Amerindians have the highest frequency of the *590A *allele with OR (odds ratio) of 3.714 (95% confidence interval - CI = 0.284-8.545; *p *= 0.040) (Table 3). Significant difference in the distribution of the *191A *allele between Amerindians and other groups was revealed by multiple logistic regression analysis, but not in UNPHASED association. The difference in results may be attributed to the small sample size of *191A *carriers among Amerindians. Moreover, the frequency of the *590A *allele in Amerindians is higher than what has been reported in studies with Amerindians from Panama (0% and 3.7% in Ngawbe and Embera Amerindians, respectively) [17,34]. Such unexpected frequency may have originated by phylogeographical differences among Amerindians populations in South-America, miscegenation, and genetic drift.

Despite Fuselli *et al*. (2007) having found that the *NAT2 *variants are homogeneously distributed across native populations, the Amerindian sample studied here showed a lower frequency of the *857A *allele (10.3%; OR = 0.391; CI= 0.053-2.898; *p*= 0.003) than those observed in two other Amerindians groups (23.3% and 22.8%) [35] (Table 3). To date, the frequency of the *857A *allele observed in this study is similar to Asian and Central America Amerindian populations [17], which corroborates the hypothesis that native Americans descend from people who migrated from Siberia thousands of years ago and therefore share their genetic background [36,37].

To elicit further information about the relationship of SNPs, a haplotype analysis was performed. Although previous studies have shown the efficiency of the PHASE method, we relied on the work of Sabbagh and Darlu (2005), which shows the effectiveness of the EM method for *NAT2 *haplotype reconstruction and suggests that there is no impact on phenotype prediction compared to results given by PHASE analysis [38]. We observed significant differences in the haplotype structure and frequency among the descendents of the three ethnic groups (Figure 1 and Table 4). Using haplotype analysis based on LD data, a haplotype block between *G590A-A803G *(Block 1; Figure 1C) was detected in Amerindians but was not found in the other two ethnic groups. This result may help to explain the highest frequency of slow acetylation haplotypes in Amerindians (Table 4). Consistent with the hypothesis that Amerindians may not be under a high selective pressure for fast metabolism, we have previously reported different distribution patterns of *GSTP1 *low activity polymorphism in this same Amerindian population [39]. Different distributions found in Amerindians, when compared with other groups, may be attributed to their low degree of admixture despite the high degree of miscegenation in the whole population. This occurs for historical reasons related to the particular way Brazil was colonized. In this way, the Amerindian group still maintains its socio-economic distinction that contributes to low degrees of admixture.

Due to our limited sample size, we suggest a careful matching of ethnicity for future larger genetic investigations. Except among the Amerindian descendents, our results suggest that self reported ethnicity might not have significant effects on the distribution of these *NAT2 *genetic variants studied in the Brazilian population. This data is relevant due to the classic role of Nat2 on isoniazid metabolism in tuberculosis treatment, which still remains an important problem of public health. In fact, several reports indicate that the acetylator status is associated with drug-induced hepatitis and *Mycobacterium*-resistance [40,41]. Furthermore, as observed in other phase II metabolizing enzyme polymorphisms, *NAT2 *genetic variants have been used as a genetic marker in different diseases like bladder and colon-rectal cancers (fast acetylator and slow acetylator, respectively) [42,43].

## Conclusions

Information gathered on the distribution of genetic polymorphism in populations of different ethnic origins remains essential to understand the interethnic differences in drug disposition and disease risk. This study demonstrates that common distributions of *NAT2 *SNPs are related with ethnic background in a Brazilian admixed population. Hereafter, DNA sequencing for the entire intron-exon organization of the *NAT2 *gene will provide more detailed information about genetic diversity and structure in this population. All these findings offer new insights for the investigation of possible non-described *NAT2 *gene-environment effects in admixed populations.

## Authors' contributions

LAVM, PRSM, GDP, and FRS designed the study. LAVM selected the subjects, collected the samples, isolated DNA, and carried out statistical analysis. JT and CVNS genotyped the samples. All authors contributed to the writing of the manuscript.
